# Supplementary material for: The Role of Lignin in the Compartmentalization of Cadmium in Maize Roots Is Enhanced by Mycorrhiza
Source: J Fungi (Basel). 2023 Aug 15;9(8):852. doi: 10.3390/jof9080852 (PMC10455880; doi:10.3390/jof9080852)
Supplement: Supplementary file 1 [file jof-09-00852-s001.zip › jof-2504783-supplementary.pdf]

Supporting information:

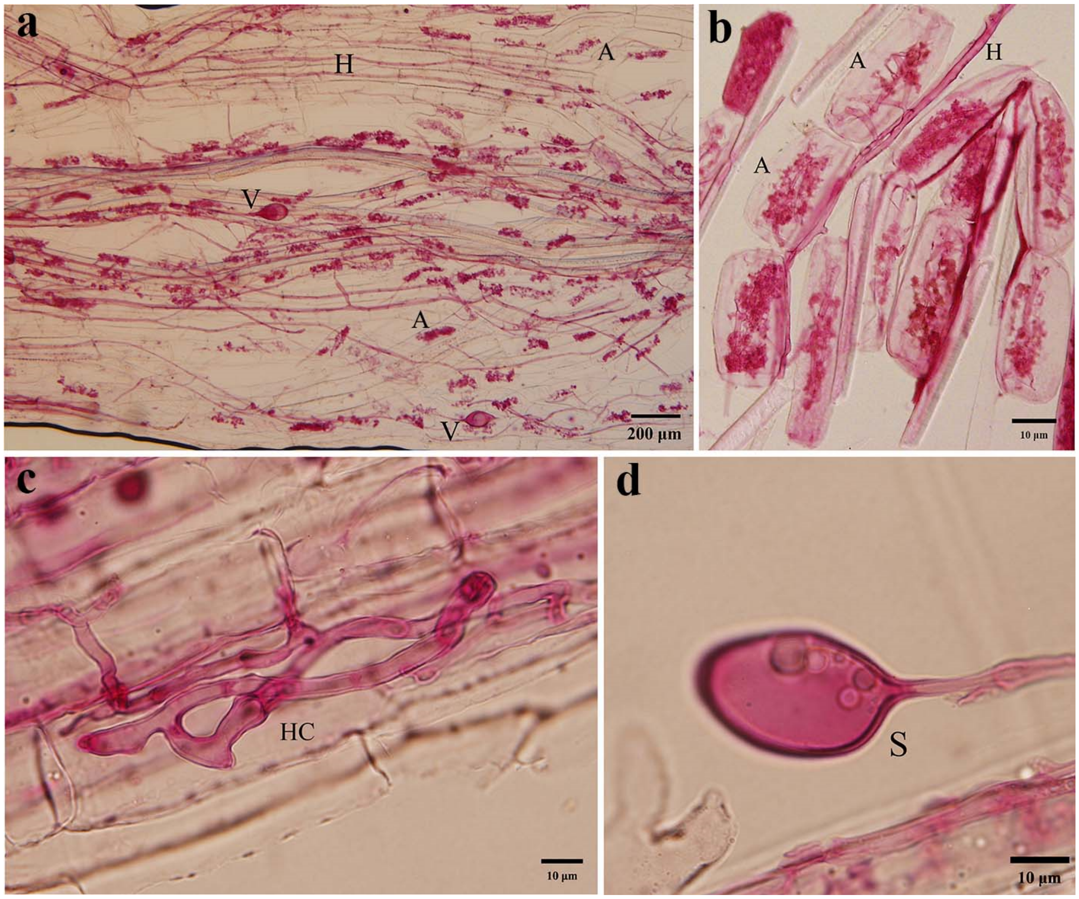

Figure S1. The typical structures of AM fungus in the roots of maize inoculated with *F. mosseae* exposed to both 0 mg kg^{-1} (Cd0) and 25 mg kg^{-1} Cd (Cd25) supplementation for a duration of 50 days are depicted in the following figures: Figs. S1a–c illustrate the presence of extensive intercellular or intracellular arbuscules (A), hyphae (H), hyphal coils (HC), and vesicles (V) in the roots of maize under the M+Cd25 condition. Fig. S1d visualizes an intracellular spore (S) colonizing the roots of maize under the M+Cd0 condition.

.

Table S1. The mycorrhizal responsive absorption peaks of functional groups (*X*) in maize root cell walls (CW) and various CW polysaccharides, including pectin, hemicellulose 1 (HC1), hemicellulose 2 (HC2), and lignin under 25 mg kg^{-1} Cd supplementation for 50 days.

NO.	Functional groups	AMF-responsive absorption peaks				
		CW	Pectin	HC1	HC2	Lignin
1	A3317 (Hydroxyl/Amino)	-0.16	0.38	-0.67	0	0.13
2	A2924 (Methyl)	0	0	0	0	0
3	A1737 (Ester group)	0.02	-0.2	0.27	-0.09	0.04
4	A1647 (Amide I)	0.1	0.12	0.07	-0.12	0.03
5	A1555 (Amide II)	0.1	0.11	-0.82	0.81	0
6	A1435 (Carboxylate)	0.03	0.03	-0.53	0.4	0.13
7	A1365 (Carboxylate)	0.01	0.01	-0.63	0.71	-0.08
8	A1255 (−C−O−S/−C−O/−C−O−P)	-0.03	-0.05	0.02	0	0
9	A1156 (Pectin polysaccharide rings)	-0.07	-0.06	-0.01	0	0
10	A1043 (C−H of Carbon chain)	-0.03	-1.36	1.36	0.03	-0.06
